# From ‘Sovereign Self‐Proclaimed Experts’ to ‘Impressionable Sceptics’–Developing a Patient Typology to Distinguish Patients' Interactions With Healthcare: A Qualitative Study in Germany

**DOI:** 10.1002/hpm.3923

**Published:** 2025-03-21

**Authors:** Katharina Achstetter, Miriam Blümel, Julia Röttger, Julia Köppen, Katherine Polin, Reinhard Busse

**Affiliations:** ^1^ Department of Health Care Management Technische Universität Berlin Berlin Germany; ^2^ Berlin Centre for Health Economics Research Technische Universität Berlin Berlin Germany

**Keywords:** Germany, health system performance assessment, healthcare, patient interaction, patient perspective, patient types, person‐centredness

## Abstract

**Background:**

Person‐centredness in health systems puts patients and their preferences at the centre of healthcare. However, there is not an ‘one size fits all’ approach as patients are heterogenous and have varying interactions with and perceptions of healthcare, and assessments of the health system performance. This study aims to explore these patient differences by (1) identifying core attributes of patients that shape their general approach to and interactions with healthcare and (2) deriving specific patient types based on these core attributes.

**Methods:**

The qualitative study included content analysis of semi‐structured, problem‐oriented interviews with 27 participants selected with the aim of maximum variation and heterogeneity (e.g., regarding age, gender, health status, place of living) from the Berlin‐Brandenburg region of Germany.

**Results:**

Based on the interviews with the participants, three core patient attributes were found that shape interactions with healthcare: (1) taking care of health and illness, (2) the self‐assigned patient role, and (3) the patient‐assigned healthcare provider role. Seven patient types were identified across (opposing) manifestations of these core attributes, ranging from ‘sovereign self‐proclaimed experts’ (focussing–autonomous–fulfiller) to ‘impressionable sceptics’ (ignoring–heteronomous–seller).

**Conclusion:**

Consideration of the identified patient types and their different ways of engaging with healthcare providers and their varying perceptions of the health system can help to develop strategies to promote person‐centredness in health systems. Furthermore, this typology can inform providers about the diverse ways in which patients may perceive healthcare interactions, and it can be useful for the training of future physicians and other healthcare professionals.

**Summary**

- This patient typology distinguishes patients regarding their healthcare interactions.
- Patients differ in their perception and assessment of the health system performance.
- Knowledge of the different patient types can be used to enhance the patient experience.
- It is useful for the training of physicians to prepare them for individualised interactions.

## 1 Background

Person‐centred health systems seek to serve population interests and put individual patients and their preferences at the centre of healthcare [1, 2, 3]. Person‐centredness can help to ‘*improve processes and outcomes as well as satisfaction*’ [4]. As the specific needs and preferences of the individual patient are put front and centre [5, 6, 7], patient‐centredness supports both the quality of care overall as well as in patient‐provider interactions. Health systems and the delivery of healthcare should thus be flexible and adept to capture and prioritise individual care preferences, expectations and needs throughout the life‐course, and seek to involve people in their own care as partners in decision‐making [8, 9]. Indeed, patients should also be empowered to participate at—and be integrated into—every level and sector of the health system to ensure its sustainability and equity, and to thereby safeguard population health [10, 11].

Patient‐centredness, including patient empowerment and shared decision‐making, is not only about healthcare quality but health system performance overall. When conducting health system performance assessment (HSPA), it is recommended to integrate the population perspective into every level and dimension (e.g., access to healthcare, quality, safety). This facilitates to identify strengths and weaknesses of a respective health system from the patient perspective and to identify performance differences for various patients [12, 13, 14, 15].

Patients differ across many dimensions, including related to sociodemographic, socioeconomic, and health‐related characteristics, and these can particularly shape their specific interactions with the healthcare system including communication with providers, their own health‐seeking behaviours and their perception of the health system [16, 17, 18, 19, 20]. Traditionally, research focuses primarily on differences based on these obvious characteristics. However, it is unclear which other concepts related to person‐centredness should be further explored to understand differences between patients in their interactions. It can be assumed that health literacy [21], self‐efficacy and patient involvement and participation in care processes [22], compliance and shared decision‐making [23] also influence individual interactions with healthcare and perceptions of a health system. Beyond these concepts, individual attitudes, experiences, beliefs or opinions [24] shape an individual's social behaviour and interactions generally and, thus, it can be anticipated that more specifically, this also affects interactions with the health system and its providers.

Additionally, the focus of research has been primarily on individual patient characteristics and concepts, with no interlinkage between them. However, Marstedt et al. (2007) emphasised that single patient characteristics do not provide sufficient information about overall patient behaviour and care preferences. Rather, patient typologies, based on types constructed from sets of characteristics, can be very meaningful and relevant to understanding patients' care‐related behaviour more completely [25]. However, most patient typologies have focused only on specific aspects of interactions with healthcare, such as the patient‐physician relationship [26, 27, 28] or communication with providers, decision‐making, and information‐seeking behaviour of patients [25, 29, 30, 31, 32]. These typologies have not yet linked patients' behaviour to their general interaction with healthcare and their perception of the health system. In this context, we sought (1) to explore and identify core attributes of patients in Germany that shape their general approach to and interactions with health, healthcare, providers, and the health system as an intermediate step, with (2) the overall aim to derive specific patient types based on the identified attributes.

## 2 Methods

The aim of this research is to explore patient attributes and identify patient types distinguishing interactions with healthcare. To capture insights into participants' experiences and subjective perceptions of their interactions with healthcare, we conducted qualitative, semi‐structured, problem‐oriented interviews with people living in the urban and rural Berlin‐Brandenburg region of Germany. The interview guideline (topics see Table 1) is rooted in relevant health services research concepts related to interactions with healthcare, such as health literacy, self‐efficacy, aspects of patient involvement (e.g., shared decision‐making, compliance/adherence), past experiences, expectations and opinions of care, patterns of health behaviour, and the overall perception of healthcare. Additionally, the interview guideline described hypothetical health‐related scenarios via vignettes to capture individuals' potential behavioural responses to certain situations. The guideline was pilot tested during the initial interviews conducted and questions were amended, refined or simplified, as necessary.

**TABLE 1:** Topics included in interview guideline.

Topic:	Questions regarding:
Opening question/experiences	(1) Description of an experience with illness (own or of a family member or friend)
Health literacy	Finding trustworthy information for different situations:
	‐ (2) Wish for examination of tinnitus
	‐ (3) Persistent stomach pain on a Saturday night
	‐ (4) Choice of hospital for elective knee surgery
	‐ (5) Long‐term care for a close family member in need
Shared decision‐making	Shared decision‐making with physician:
	‐ (6) Pain medication versus physiotherapy for treatment of back pain
	Dissatisfaction with and switching of:
	‐ (7) Physicians
	‐ (8) Health insurance
Self‐efficacy	(9) Promoting health in everyday life
Compliance/adherence	(10) Reaction to physician's recommendation or advice
Opinion and attitude	(11) Attributes of good healthcare (and good physicians)
	(12) Overall opinion about the German healthcare system (including good components and room for improvement)
Closing question	(13) Additional comments

### 2.1 Study Sample

We applied a purposive sampling strategy to recruit participants in the Berlin‐Brandenburg region of Germany, with a focus on maximum variation [33] to capture multiple perspectives and to reach representative diversity and heterogeneity of the participants regarding sociodemographic and health/healthcare‐related characteristics, namely age, gender, chronic disease, type of health insurance, place of living, employment status, and number of physician consultations in the past year. We used multifaceted recruitment methods, including placing notices on blackboards of supermarkets and pharmacies and advertising in local newspapers and popular German classified websites. The Berlin‐Brandenburg region was chosen because of its urban‐rural mix and due to accessibility for the researchers. Participation in the study was voluntary and a reward of 25€ was used as an incentive to participate. Interviews were conducted between January and May 2017. One of five researchers (four females and one male) conducted each interview in a quiet and private setting, either in a conference room at the researchers' institution or at the participant's place of residence, depending on preference. All researcher conducting the interviews (four of the authors—KA, MB, JR, JK—and one further researcher—JMF) had a background in public health, medicine, or sociology and previous experience conducting qualitative interviews. None had a personal relationship to the participants. Each participant was informed about the research aim but was not given personal information about the researcher to maintain neutrality and avoid bias or social desirability. Written informed consent was obtained from all participants prior to the interview and interviews were audio‐recorded. Field notes were taken by the researchers during and/or after each interview. Transcription was conducted verbatim by an external transcription service and subsequently proofread by the researchers for quality assurance; interviews were then anonymised.

### 2.2 Data Analysis

Interviews were analysed using qualitative content analysis [34], identifying deductive and inductive categories through an iterative process until theoretical saturation was achieved, that is new codes were no longer identified. Deductive categories were developed, informed by the concepts and constructs used to structure the interview guideline, and inductive categories were derived from the analysis. Three researchers performed coding using the software ATLAS.ti. Each transcript was coded separately by at least two researchers. Discrepancies in *coding* were solved through discussions among the three researchers to reach a consensus. Ultimately, the deductive and inductive categories were consolidated by clustering, and reduced to core patient attributes, along with their manifestations. Manifestations were defined as the most extreme imaginable opposites for each of the core attributes.

The definition of final core attributes and their manifestations was followed by the construction of patient types. While patient types are distinguished in the manifestations of each attribute (external heterogeneity), manifestations within a certain patient type resemble each other (internal homogeneity) [35]. Participants were clustered according to the combinations of their core attributes' manifestations. These clusters were then abstracted and described as patient types. This approach to constructing a patient typology corresponds to recommendations by Kluge [35] and Kuckartz [36].

### 2.3 Ethics and Reporting

This study is part of the larger project ‘IPHA’ (Integrating the Population Perspective in Health System Performance Assessment), which aims at undertaking a comprehensive performance assessment of the German health system from the population perspective based on the intermediate and final goals defined in the World Health Organization's Health Systems Framework [14, 15] and to identify differences in this assessment among the population, respectively patient types [37].

The study was approved by the ethics committee of the Technical University of Berlin (BU_07_20160926). Study methods are reported according to the consolidated criteria for reporting qualitative research (COREQ) [38]. All presented quotations were translated from German to English. To ensure rigour, they were also translated forward and backward.

## 3 Results

### 3.1 Interviews

In total, 27 individuals were interviewed, and all interviews were completed and included in the analysis. The participants varied greatly in terms of age, gender, whether they have a chronic disease, education level, employment status, health insurance type, residence type, and use of healthcare within the last 12 months (see Table 2). Most participants were female (59.3%), with at least one chronic disease (63.0%), had statutory health insurance (92.6%), lived in an urban region (66.7%), and had on average 11.3 physician encounters in the previous year (includes all types of physicians and/or dentists).

**TABLE 2:** Participant characteristics.

Characteristics	*n*	%
Participants	27	100
Age (years)		
Mean (SD)	47 (15.1)	
Median/Range	52/18–77	
Gender		
Female	16	59.3
Male	11	40.7
Chronic disease		
Yes	17	63.0
No	10	37.0
Health insurance		
Statutory	25	92.6
Private	2	7.4
Place of living		
Urban	18	66.7
Rural	9	33.3
Physician consultations within past 12 months (number)		
Mean (SD)	11.3 (10.7)	
Median/Range	8/0–40	
Highest level of education (duration in years)		
None/declined to answer	1	3.7
School‐leaving certificate (9 years)	5	18.5
Intermediate school‐leaving certificate (10 years)	4	14.8
Advanced technical college certificate (12 years)	3	11.1
Advanced school‐leaving certificate (12–13 years)	14	51.9
Highest level of vocational qualification		
None/declined to answer	3	11.1
Apprenticeship	11	40.7
Technical/Vocational school	4	14.8
University degree	9	33.3
Employment status		
Full‐time	7	37.0
Part‐time	5	18.5
Studying	3	11.1
Retired/not employed	10	25.9
Other/declined to answer	2	7.4
Duration of interview (minutes)		
Mean (SD)	42:49 (16:50)	
Median	37:57	
Range	24:40–87:41	
Place of interview		
Participant's place of residence	8	29.6
Research institution	19	70.4

Abbreviation: SD–standard deviation.

### 3.2 Core Patient Attributes

**FIGURE 1:**
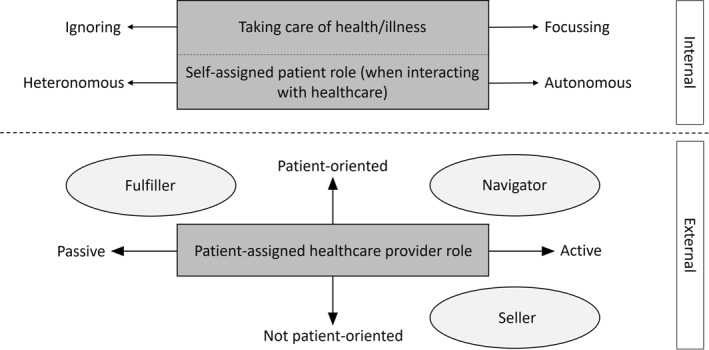
The three identified core attributes with their manifestations.

Through the analysis, three data‐driven core patient attributes (two internal, one external) emerged, as an intermediate step for the subsequent patient typology. Internal attribution denotes a tendency for patients to ascribe responsibility for their behaviour to themselves, their abilities, their character traits and their performance. In contrast, external attribution implies that patients perceive external factors, such as other individuals, circumstances, the wider environment or chance, as the motivating forces behind their actions. The three core attributes (see Figure 1) cover: (1) taking care of health/illness (ranging from a strong focus on health/illness in everyday life to ignoring health/illness) and (2) self‐assigned patient role when interacting with healthcare providers (ranging from autonomous to heteronomous), and (3) the patient‐assigned healthcare provider role (ranging from active to passive and between patient‐oriented and not patient‐oriented).

1. Taking care of health/illness: focussing versus ignoring

Focussing on health/illness implies that health/illness is perceived by participants to be a major aspect of everyday life and of which active management is a strong determinant for quality of life. This can mean, for example, that efforts to maintain or restore health were made, but also that a poor health status was perceived as a strong limitation in life if no further actions such as attempts to restore health were undertaken. Focussing on health/illness was often accompanied by a largely open mind to different forms of treatment (e.g., complementary and alternative medicines). The ‘objective’ or clinical seriousness and/or severity of an individual's health status was not necessarily related to the level of focus giving to health.

Health is the essential thing. Yes, nothing is more important than that. (P24)

So, there are many things [I do for my health in everyday life]. So, on the one hand, I am very conscious, very health conscious. That's very, very important. One thing is, that I think about what food is good and what I drink, that's important too. […] The second [thing] is, I do a lot of sports. […] So, I'm doing everything [what is good for my health]. (P24)

Ignoring health/illness was seen as a behaviour comprising very few health‐related actions. Illness and disease‐related symptoms were rarely considered in everyday life and therefore had only a minor impact on the individual's lifestyle choices and health‐related decisions.

I don’t [do anything for my health], no. I could actually do like this, where you move your arms like this, in the evening for example now and then and so on, I could do this, but then somehow, I don't feel like it. I don't know why. (P7)

[The physician] has recommended a […] diabetes school, where I should attend four appointments […] but until now, I didn’t go there. (P9)

2 Self‐assigned patient role: autonomous versus heteronomous

An autonomous role was characterised by a strong assertion of independence, the perceived ability and especially the wish to make own decisions. While interacting with healthcare providers, self‐assigned autonomous individuals preserved responsibility for their own health. The autonomous patient was often prepared for every interaction with a provider, for example, by collecting information ahead of the encounter. Interviews showed that the autonomous self‐assigned patient role was often shaped by an individual's social network (e.g., having a network of social contacts working in the health sector, especially family members), and more autonomous interactions were seen among participants with higher self‐efficacy.

I have decided that for myself. The doctor doesn’t know that, but I decided that for myself. […] So, to say, I prescribed it myself. (P25)

Patients with a heteronomous self‐assigned role were often dependent and relied on others, for example, a family member or provider, for decision‐making support regarding their own health. They tended to be directed by others and delegated most responsibility to healthcare providers.

And [the specialist] usually recommends me [to someone]. He tells me to go here or there, and I'll do that. (P10)

3 Patient‐assigned healthcare provider role: fulfiller, navigator, and seller

This patient‐assigned role of healthcare providers emerges on two axes, between the poles active and passive and between patient‐oriented and not patient‐oriented, and can be described by three manifestations: ‘fulfiller’, ‘navigator’, and ‘seller’ (see Figure 1).

Providers as a ‘fulfiller’ were expected to only react to a patient's wishes. From a patient's perspective, the purpose of interacting with a provider was to fulfil a desire/request (e.g., for prescription medication) or the patient was seeking approval of own decisions. Further suggestions or any kind of active engagement from the provider were mostly unwanted.

So, for me, a doctor is someone who makes a diagnosis, which is normally the one I have already [made], so for me it's just a confirmation. (P6)

The patient‐assigned healthcare provider role of ‘navigator’ carried expectations of a typical case manager with a positive patient‐provider relationship in a well‐balanced partnership. Decisions were made on an equal basis through discussing different options, negotiating what would be best, and finally a joint decision.

So, in any case a [good] doctor [is someone], who listens to me well and who really looks individually, who looks closely, who is not superficial, who really looks closely and also listens closely to what I say. […] I also get up and leave when I notice I don't feel properly cared for. That has already happened, that I have also discontinued a therapy because I was not taken seriously. So really listening actively and then deciding together with me individually what is the right way for me. That constitutes a good doctor. (P24)

The role of ‘seller’ was when the patient perceived the provider to have fraudulent intentions or ulterior motives often in opposition to the well‐being (e.g., selling products, earning money, supporting colleagues). Participants felt suspicious of a recommendation made by the ‘selling’ provider.

First of all, doctors want a lot of money, […] so to speak, so I always feel like they persuade you into extra stuff, so they can get the money. (P1)

### 3.3 Patient Types

Seven patient types based on different combinations of the core attributes' manifestations were identified among the participants:

Type 1: ‘Sovereign self‐proclaimed expert’ (focussing–autonomous–fulfiller).

The ‘sovereign self‐proclaimed expert’ focuses strongly on health in everyday life, taking good care of health/illness, has broad knowledge of relevant health‐ and illness‐related topics and is very well‐informed about how to maintain or promote health in general. This type acts very independently and is not willing to share responsibility for health/illness with others. This type looks to providers only to fulfil requests and not for engagement and shared decision‐making and thinks to be the best expert for their own wellbeing. Participants forming this type tend to actively self‐care and to treat themselves without consultation of a provider (self‐treatment).

First of all, I don't trust anyone, but I collect [information] […] from these three [friends, physicians, and the internet] and then I decide on my own. (P23)

Type 2: ‘Well‐informed equal partner’ (focussing–autonomous–navigator).

The ‘well‐informed equal partner’ also strongly focuses on health/illness in everyday life. This type perceives itself to be autonomous but is willing to interact on an equal basis with providers and to participate in shared decision‐making. Type 2 prefers a balanced partnership with providers, where each side's knowledge and experiences are respected.

Well, I have to feel, yes, he [the physician] understands what I am talking about […] [because] I also think about it myself and he has to go in the same direction. I must feel that. And then I have trust (P24)

Type 3: ‘Sovereign sceptic’ (focussing–autonomous–seller).

The ‘sovereign sceptic’ has a high degree of autonomy and focuses on the own health/illness. This type shows a high affinity for self‐care and is characterised by suspicion of providers and their intentions. From this type's point of view, most providers only want to sell their products, that is, healthcare services, with the aim of earning money or furthering their own interests above the wellbeing of their patients. As such, the ‘sovereign sceptic‘ perceives to be used and exploited by providers.

[A]mong my friends, among my acquaintances, I also have physicians and also my dentist, with whom I am friends, and he has also said that it is quite often actually already assault, what his colleagues do and only in order to earn money. (P15)

Type 4: ‘Compliant partner’ (focussing–heteronomous–navigator).

The ‘compliant partner’ focuses on health/illness and looks for joint health decision‐making with providers. However, this type assigns itself a heteronomous role in this relationship, and not only seeks advice but looks to strictly follow healthcare provider guidance. In this partnership, the person ultimately making the decision is the provider.

I am a person in that respect who listens very much to the doctor. Because he has studied, that is his specialty, he is competent, and I trust the doctors basically. (P17)

Type 5: ‘Avoidant partner’ (ignoring–autonomous–navigator).

The ‘avoidant partner’ ignores everything related to health/illness in everyday life regardless of health status. This type's self‐assigned patient role is characterised by perceived balanced partnerships with providers, who serve as navigators. While provider recommendations are taken seriously, because of this type's passive, avoidant way of thinking about and dealing with health/illness, they are rarely put into practice if at all.

No, actually I don't do anything [for my health] actually. I could actually do this, lie down and massage a bit or go up and down with my body so that the body is massaged a bit. But to be honest, I don't feel like it or anything. (P7)

Generally, I don't go to the doctor very often, unless it's absolutely necessary. So, I really only do those things where I know I have to do them now. (P4)

Type 6: ‘Impressionable sceptic’ (ignoring–heteronomous–seller).

The ‘impressionable sceptic’ mostly ignores thoughts of health/illness in everyday life, regardless of health status. This type is influenced by others (e.g., friends and family members), is sceptical of healthcare providers (‘seller’) and primarily driven by opinions/recommendations of others whom this type trusts.

I fell and broke my arm, and my mother said that I do not need to go to the doctor for now and so I only went to the doctor after a week. (P1)

Type 7: ‘Submissive partner’ (ignoring–heteronomous–navigator).

The ‘submissive partner’ largely ignores health/illness, despite health status. Additionally, this type does not take responsibility for health‐related decisions and looks to others for guidance through all health‐related decisions. The ‘submissive partner‘ views providers as navigators to be followed unquestioningly; all actions and changes of behaviour are driven and initialised by providers and not happening out of the internal motivation.

I always need someone to take me along a little bit, you know, but apart from this, there's nothing [no ambition to do something for my health] by itself. (P10)

**FIGURE 2:**
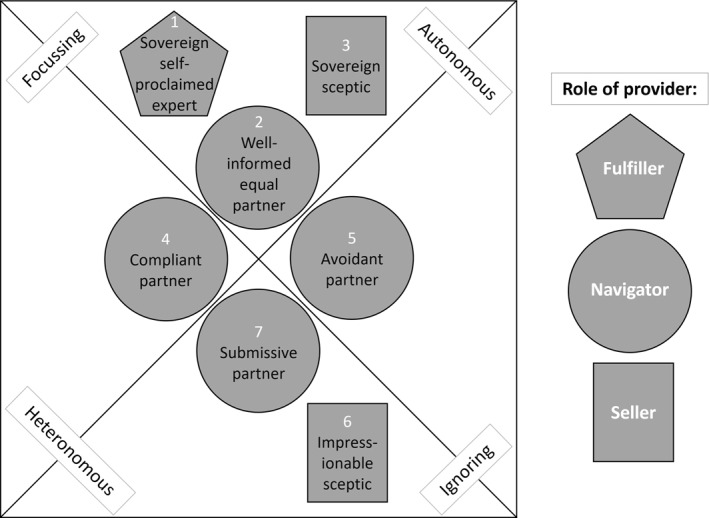
Clustered patient types according to their attributes' manifestations.

Figure 2 clusters the seven identified patient types according to the combinations of the attributes' manifestations. Each manifestation of the internal attributes (focussing, ignoring, autonomous, heteronomous) is represented by three or more patient types, whereas the manifestation ‘fulfiller’ of the external attribute is only represented by the “sovereign self‐proclaimed expert”. The provider as navigator informs the most patient types (four out of seven).

## 4 Discussion

The objectives of this study were to (1) identify core attributes that influence a patient's interaction with healthcare and providers and the perception of the health system, and (2) derive patient types based on these identified attributes to construct a patient typology. Twenty‐seven interviews were conducted with heterogeneous patients in the Berlin‐Brandenburg region of Germany. Three core patient attributes with two or three manifestations each emerged from the analysis, namely, to what extent a patient pays attention to health/illness in life; the self‐assigned role in the healthcare decision‐making process; and the role the patient assigned to the provider. Ultimately, seven distinct patient types were identified ranging from ‘sovereign self‐proclaimed experts’ (focussing–autonomous–fulfiller) to ‘impressionable sceptics’ (ignoring–heteronomous–seller).

Taking care of health and illness is a central part of the individual's health behaviour, which has a direct impact on the health status [19, 39] and is influenced by characteristics such as social status [40], social support [41, 42], fears and wishes [18], individual motivation, self‐efficacy [43], beliefs and convictions [44], and health literacy [21]. Promoting, for example, health literacy and self‐efficacy can therefore support people to focus on health and take active care of their health and illness in everyday life. According to a representative survey (*n* = 4808) in Germany in 2010, health was subjectively the most important area of life, regardless of age, gender, and social class [45]. An individual's subjective assessment of the importance of health is also influenced by the level of perceived control over the own health as well as the perceived achievability of self‐defined health goals [46].

Previous research looking at preferences for participation in healthcare decision‐making also distinguished patients as either ‘autonomists’ who control all decisions and ‘delegators’ for whom the physicians should decide [29]. According to Dierks and Schwartz [47], patients differed in their roles between passive and active and between cooperative and sovereign‐independently. These roles are comparable to the self‐assigned patient roles (autonomous vs. heteronomous), combined with the patient‐assigned healthcare provider role (especially the ‘navigator’ and the ‘fulfiller’).

From the interviews, it became apparent that the degree of autonomy in the self‐assigned patient role is influenced by other factors such as a patient's social network, particularly participants working in the health sector or those with close family members with health‐related jobs presented an autonomous self‐assigned patient role. It could be assumed that higher education and high self‐efficacy were preconditions for having an autonomous self‐assigned patient role and for pursuing shared decision‐making in interactions with providers, which should be a subject for further research.

The interviews showed that autonomy was linked with a greater tendency towards self‐care. Promoting self‐care empowers patients, supports informed decision‐making and, therefore, even contributes to improving efficiency of the healthcare systems and health equity [48]. Hence, supporting autonomy in patients could be helpful for strengthening the healthcare system, promoting population health, and improving the overall performance of a health system.

The patient‐assigned healthcare provider roles ranged between ‘fulfiller’, ‘navigator’, and ‘seller’. Schmöller [49] differentiated physicians into ‘value‐conservative paternalists’, ‘patient‐oriented partners’, ‘customer‐oriented service providers’, and ‘liberal autocrats’. These concepts can be partially compared to the provider as ‘navigator’ and ‘fulfiller’ in our study. Other studies had also pointed out that the physician‐patient interaction and physicians' treatment decisions can be driven and motivated by financial aspects [50, 51, 52]. According to Röttger et al. [53], trust in a physician includes whether a physician is patient‐oriented or guided by other interests. This is in line with the identified patient‐assigned healthcare provider role of a ‘seller’, where the provider pursued interests beyond the patient's well‐being.

It can be assumed that the identified core patient attributes and the patient types were in part a result of the patients' experiences, social environment and status, and shaped by the previous interactions with the healthcare and healthcare providers. It must be considered that while most patients seemed to be attributing the same role to all healthcare providers, there could be cases in which different roles were assigned to single providers. Additionally, these patient‐assigned healthcare provider roles were not rigid or fixed and could change with further experiences and evolve over time. Provider interactions described in the interviews mostly related to experiences with physicians, while interactions with other types of providers were not explicitly asked about, and not every patient has had experiences with other healthcare professionals (e.g., nurses, physiotherapists). This should be subject to further research to inform each profession/healthcare provider about emerging interaction types for respective fields.

In general, it is unclear how the distribution of patients across the seven patient types occurs in real‐life healthcare and if they are equally present. Further research could try to map day‐to‐day interventions of providers in this typology with the aim of highlighting the most ‘common’ patient types. It can be further assumed that particularly the ‘extreme’ patient types regarding their provider‐assigned roles (i.e., type 1, 3 and 6) are more challenging for providers in their interactions and therefore require specific attention and more tailored forms of interaction. More generally, patient types containing the ‘ignoring’ attribute (types 5–7) need more external focus on their health and healthcare, while heteronomous patients (type 4, 6, and 7) benefit from a strong guidance of their provider. Focussing (types 1–4) and autonomous (types 1–3 and 5) patient types could be considered more straightforward patients in their interactions with providers. This could be subject to further research to develop recommendations what works best for each of the patient types and to test and verify the effectiveness of tailored interaction and communications styles.

A more detailed look into individual patient types reveals more distinctive aspects for tailoring suitable patient‐provider relationships. For example, type 1, the ‘sovereign self‐proclaimed expert’ (focussing ‐ autonomous ‐ fulfiller) reduced provider interaction to a minimum, made own decisions and looked to providers only to fulfil self‐identified needs or requests. This type is also very attentive and tries to discern when and why an illness may have occurred, what exacerbates and what helps it and seeks information from the social network (e.g., family members working in the health sector) and the internet. This highlights the necessity of having reliable information sources particularly for this patient type who is seeking information about their health/illness, particularly prior to an interaction with a healthcare provider. This type requires not much guidance from a provider but could benefit from a ‘peer‐like’ role to jointly and critically assess the information retrieved by the patient. Providers can probably interact best with these patients when interacting on eye‐level and negotiating as equal partners, while giving the patient the ultimate decision power. Therefore, it can be assumed that these patients will highly value providers asking them about their ideas, wishes, and suggestions during each interaction.

As another example, type 7, the ‘submissive partner’ (ignoring, heteronomous, navigator) was very dependent and relied on a provider to focus on health/illness‐related problems and decisions. These patients would ignore illnesses and would only communicate symptoms if asked directly. For all health‐related decisions, this type needs someone for advice and guidance. Therefore, the ‘submissive partner’ strongly relies on providers as decision‐makers and would benefit from gatekeepers or case‐managers. Being aware of the needs of this patient type would allow providers to take on this role and ensure guidance during the care pathways of respective patients. Therefore, this type would presumably benefit from a strong hands‐on interaction type with a clear provider‐predefined patient pathway illustration. Each step within the care process and every single healthcare measure (e.g., check‐ups, medication) need to be strongly emphasised. This could be carried out through for example, a tailored checklist combined with regular reminders, via digital health applications, mail or telephone depending on the patients' and/or providers' preferences.

## 5 Conclusion

Patient interactions with healthcare, providers and the perception of the health system were seen to be informed by distinct patient types. Subsequently, future person‐centred approaches should consider these patient types, including their underlying attributes, similarities, and differences. Knowledge and understanding of these patient types can also help to strengthen and tailor interventions for specific patient types and improve communication and patient‐provider partnerships. The results of this study are highly relevant for healthcare providers to understand the various encounters they have with patients every day. They can thus be useful for the training of future physicians and other healthcare providers, and to prepare them not only for interactions with different patient types, but also for managing ongoing relationships.

Moreover, this study can also support the implementation of the concept of person‐centredness within all sectors of healthcare and the health system. Person‐centredness in health systems has, to date, been highly aggregated and needs to be disaggregated for the different patient types to be able to identify and analyse inequities in systems and to design and implement appropriate and just interventions. Putting an entire population and its mass of individual preferences at the centre of the healthcare system requires also considering and unpacking the diversity and variety of all the component persons. This study's identified patient types with their diverse ways of engaging with the healthcare system and healthcare providers can support evidence‐based policymaking and help to develop different strategies to promote person‐centredness. Additionally, further interpretation and discussion about the implications that these patient types and their differences have on the perception of healthcare and the assessment of the health systems' performance, for example, on the access to and quality of healthcare, is still much needed.

## 6 Limitations

Despite this study's overall strength and innovation, some limitations need to be considered. These are due to the overall recruitment strategy of the participants and include geographic limitations (only participants from Berlin‐Brandenburg region in Germany were included), recruitment‐based limitations (only those who read the call for participation in certain outlets were included), interest‐based limitations (only persons with a strong interest in health‐topics were willing to participate), and language‐based limitations (call for participation was only in German). Furthermore, it is possible that the employment of an external incentive led to persons only participating because of the monetary reward, leading to limitations in the study sample. Based on the results, it can be assumed that especially patients with more experience of interacting with the healthcare system and with a broad knowledge of health topics participated in this study. In addition, face‐to‐face interviews with a professional researcher required a certain amount of self‐confidence to follow the call for participation. In addition, there might have been a social desirability bias in participants' recounting of their experiences, opinions, and knowledge of the health system, with some participants attempting to give more desirable or expected answers than their true beliefs/opinions.

## Author Contributions

K.A. had the main role in manuscript preparation, supported by M.B., J.R., J.K., K.P. and R.B. R.B. and M.B. were primarily responsible for conceptualising and developing the overall project and study design. K.A., M.B., J.K. and J.R. led on data collection and analyses. All authors were responsible for reading, commenting upon, and approving the final manuscript.

## Ethics Statement

The study was approved by the ethics committee of the Technical University of Berlin (BU_07_20160926). All study participants provided informed written consent prior to study participation.

## Conflicts of Interest

The authors declare no conflicts of interest.

## Data Availability

The datasets generated and analysed during the current study are not publicly available due privacy/ethical restrictions but are available from the corresponding author on reasonable request.
